# Neck-specific exercises with internet-based support compared to neck-specific exercises at a physiotherapy clinic for chronic whiplash-associated disorders: study protocol of a randomized controlled multicentre trial

**DOI:** 10.1186/s12891-017-1853-1

**Published:** 2017-12-12

**Authors:** Anneli Peolsson, Maria Landén Ludvigsson, Gunnel Peterson

**Affiliations:** 1 Department of Medical and Health Sciences, Physiotherapy, Linköping University, Sandbäcksg. 7, 58183 Linköping, Sweden; 2 Department of Rehabilitation and Department of Medical and Health Sciences, Rehab Väst, County Council of Östergötland, Linköping University, Linköping, Sweden; 3 Centre for Clinical Research Sörmland, Uppsala University, Eskilstuna, Sweden

**Keywords:** Whiplash injury, Neck, Spine, Chronic, Physiotherapy, Rehabilitation, Exercise therapy, Randomized, Follow-up study, Outcome

## Abstract

**Background:**

Globally, neck pain is the fourth most common condition associated with longer periods of living with disability. Annually, approximately 0.3% of the population of Western countries undergo whiplash trauma, and half of those individuals will develop chronic problems with high costs for the individual and society. Evidence for chronic whiplash-associated disorders (WAD) treatment is scarce, though neck-specific training at a physiotherapy clinic twice a week for 12 weeks has demonstrated good results. More efficient, flexible rehabilitation with reduced waiting times and lower costs is needed, ideally replacing lengthy on-site treatment series by healthcare providers. Internet-based care has been shown to be a viable alternative for a variety of diseases and interventions, but studies are lacking on Internet-based interventions for individuals with chronic neck problems. The aim of the trial described here is to compare the effects of an Internet-based neck-specific exercise programme to the same exercises performed at a physiotherapy clinic in regards to self-reported and clinical measures, as well as cost-effectiveness.

**Methods:**

This prospective, randomized controlled trial will involve 140 participants. Measurements will be made at baseline, 3 months (end of treatment), and 15 months (12 months after end of intervention) and will include ratings of pain, disability, satisfaction with care, work ability, quality of life, and cost-effectiveness.

**Discussion:**

The study results may contribute to the development of a more effective rehabilitation, flexible and equal care, shorter waiting times, increased availability, and lower costs for healthcare and society.

**Trial registration:**

ClinicalTrials.gov Protocol ID: NCT03022812, initial release 12/20/2016.

## Background

### Epidemiology, aetiology, cost, and symptoms

Of 301 classified diseases, neck pain is fourth in the “Global Burden of Disease Study” [1], which evaluated years lived with disability. A common reason for neck pain is a whiplash injury, with an annual reported incidence of approximately 0.3% [2, 3]. Whiplash trauma is an indirect neck trauma in which acceleration, deceleration, and the compressive forces of the head are transmitted to the cervical spine, exposing it to high mechanical forces during the whiplash movement. Of those who are injured, roughly half experience chronic (≥6 months) WAD [4–6]. Whiplash injury is graded in five degrees according to the most used classification, the modified Quebec Task Force (QTF) [7]. Common symptoms in WAD in addition to neck pain are headaches, radiculopathy, dizziness, balance problems, mental illness, sleep disorders, lower quality of life, low general health, and a decreased ability to work [2, 4, 6, 8–11], leading to very high individual and social costs [2–4].

### The knowledge gap

Strong evidence is lacking on how chronic WAD should be treated [12–16]. Although exercise has the best quality evidence [12, 13, 16], previous studies of exercise in chronic WAD have shown somewhat disperse results. Considering the chronicity of the population, large group differences cannot be expected, but smaller clinically relevant improvements can be very valuable. In WAD grade 1–2 (grade 1 = no physical findings; grade 2 = local neck findings), simple advice is equally as effective as a more intense and comprehensive physiotherapy exercise programme [17]. However, in WAD grade 2–3 (grade 3 = grade 2 + neurological findings), neck-specific exercise is more effective than prescribed physical activity 2 years post-treatment [10, 11, 18–21]. One reason for the different results may be the different WAD grades, as WAD grade 3 has been associated with treatment success [18, 19]. It is also reasonable that people without physical signs of WAD and only self-reported pain (grade 1) will do just as well with advice. Another explanation may be that different kinds of exercise programmes have been used that often included only elements of exercises targeting the neck muscles [17, 22, 23] or improved aerobic capacity without neck-specific exercises [24]. The main outcome also varies among studies. Information on the effect of neck-specific exercise programmes consisting of both neuromuscular and neck muscle endurance exercises for both ventral and dorsal neck muscles is scarce, as are studies involving patients with higher WAD grades (grade 3) [18, 19]. Studies regarding physical exercise in individuals with chronic pain, WAD and WAD-related headaches included [15, 25–27] has been identified as representing priority knowledge gaps.

The currently planned study would help fill these gaps and be important for future rehabilitation, with benefits for both individuals and society. For individuals with chronic WAD, studies on Internet-based care are lacking. Similarly, no study has included specific neck exercises distributed through the Internet for individuals with neck problems.

### Why is it important to exercise the neck muscles?

The term whiplash does not communicate the actual pathology, only the mechanism of injury. Although the aetiology and diagnosis regarding WAD are unclear, some of the symptoms can be related to various cervical spine structures, such as the muscles, ligaments, facet joints, discs, and nerves [28–33], leading, for example, to inflammation, fat infiltration, muscle fibre conversion, lower range of neck motion, disturbed neuromuscular control, and impaired neck muscle function [34–45]. Indications also exist for disturbed muscle function predisposing an individual to recurrent problems [46].

The muscle function of the cervical spine is very important for stability, eye–hand coordination, postural control, and interactions between the vision and vestibular systems for good balance and to avoid dizziness, pain, and disability [47, 48]. Deteriorated muscle function does not seem to be restored automatically without specific neck exercises [34, 46, 49–51]. For individuals with mechanical neck pain, including WAD, evidence suggests that neck exercise is effective [14, 52–55], and it is recommended in clinical guidelines [56]. However, most intervention studies involve acute WAD and research addressing neck-specific exercises for both ventral and dorsal neck muscles, consisting of both neuromuscular and endurance exercises, in chronic WAD is scarce. Only Ludvigsson et al., Landén Ludvigsson et al. [18, 19], and Peolsson et al. [57] included individuals with more severe WAD (grade 3) to compare three exercise interventions: neck-specific exercise, neck-specific exercise combined with behavioural therapy, and prescription of general physical activity. When the interventions were completed after 3 months of training (2 times/week, total of 24 times), the individuals who practised neck-specific exercises improved, with no significant differences between the two neck-specific groups, and the general physical activity group remained almost unchanged or worse [10, 11, 18–20]. In another study, neck-specific exercise for chronic WAD was compared to being on the waiting list for 3 months [57]. The neck-specific exercise group improved as the waiting list group deteriorated in most variables [57]. Neck-specific exercise is recommended for individuals with chronic WAD [16], but further research is needed, especially for those with chronic WAD and greater disability.

### E-health

The healthcare system is undergoing challenges of the future; care needs to be developed, improved, and made more efficient while also offering increased availability and controlled costs. Patients of working age can also have difficulty taking time off from work to access the provided healthcare. It is also important to gain access to care despite geographical distances. New ways of offering healthcare need to be developed to increase availability and patient adherence, shorten waiting times, and reduce costs, especially in the case of longer treatment series, as with exercise treatment at a physiotherapy clinic for those with chronic problems. Internet-based treatment may also develop and strengthen the patient’s own resources for greater autonomy and the ability to conduct long-term self-care [58]. Internet-based healthcare has been increasing as technology evolves and more individuals have access to a computer/tablet/smartphone and the Internet [59]. This kind of treatment delivery could also facilitate the implementation and deployment of therapy shown to be effective in research studies [10, 11, 18–20]. Treatment delivery via the Internet has good, equal, or better efficacy than face-to-face treatment with the caregiver for a number of diseases, including exercises for individuals with stress incontinence, low back pain, heart disease, rheumatoid arthritis, and after knee arthroplasty, as well as for chronic pain [60–65]. People report preferring Internet-based delivery because of the increased flexibility and convenience [66, 67]. However, disadvantages of relying only on Internet-based care include insufficient support, information, and understanding. A few visits to the caregiver combined with Internet-based support has been shown to be preferable [68].

Regarding people with neck problems, including WAD, only one study has incorporated Internet-based care [69]. Bring et al. [69] included individuals with acute WAD grade 1 and 2 and reported that a behavioural programme of direct encounters with the healthcare provider and an Internet-based behavioural programme both led to less pain-related dysfunction, and both were better than the standard treatment (i.e., a pamphlet). However, for individuals with chronic WAD, including those with more severe degrees of WAD (grade 3), studies on Internet-based care are lacking. No study has included specific neck exercises distributed through the Internet for individuals with neck problems. Furthermore, studies evaluating the comparative costs of these modes of rehabilitation are also lacking.

Although previous studies on chronic WAD (10, 11, 18–21, 57) reported that 12 weeks of neck-specific exercise is effective and cost-effective, it requires quite a bit of resources. Thus, it is important to investigate whether the same effect can be achieved if delivered in a more cost-effective way with fewer physiotherapist visits and an Internet support.

### Aim

The aim of the trial is to compare the effects of an Internet-based neck-specific exercise programme (NSEIT) with the same exercises (NSE) performed at a physiotherapy clinic in regards to self-reported and clinical measures, as well as cost-effectiveness. Further objectives are to identify factors associated with the outcome following exercise regarding pain and disability.

Hypothesis: Internet-based neck-specific exercises with four visits to the physiotherapist will be non-inferior to neck-specific exercises at a physiotherapy clinic twice a week for 12 weeks (i.e., treatment NSEIT is not inferior to NSE) at 3- and 15-month follow-ups, and NSEIT yields lower economic costs for society than NSE.

## Methods/design

### Design

**Fig. 1:**
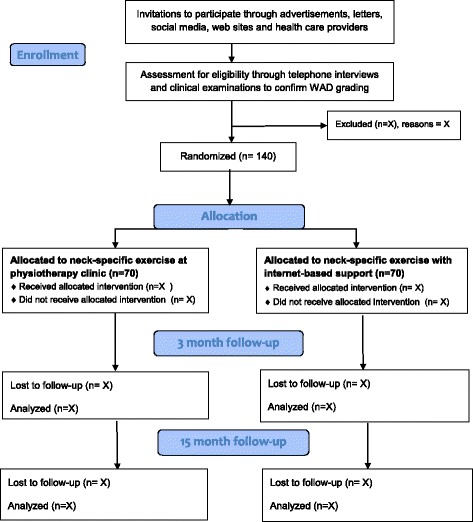
CONSORT Flow Diagram for Neck-specific exercises with Internet-based support compared to exercises performed in a longer series at a physiotherapy clinic: a prospective randomized multicentre study of people with chronic whiplash-associated disorders

This is an experimental, longitudinal, prospective, multicentre, randomized controlled trial (RCT) with two parallel treatment arms conducted according to a detailed protocol decided on before recruitment started (ClinicalTrials.gov Protocol ID: NCT03022812). Physiotherapist-led neck-specific exercise previously shown to be effective for the current population [18, 19] constitutes the control treatment for the new Internet-based neck-specific exercise treatment. A total of 140 people (70 in each group) are expected to be included, mainly by self-selection after advertisements. Independent physiotherapists in primary care will distribute the treatment. Due to the nature of the study, neither participants nor treating therapists can be blinded. To be un-biased to data the project manager will not be involved in the data collection. The physical measurements will instead be performed by independent test-leaders/specially trained, skilled, physiotherapists blinded to randomization. Data collection in the form of questionnaires and tests of physical neck-related function occurs at baseline (before randomization) and after 3 (end of physiotherapy rehabilitation) months and 15 months (1 year post-intervention) (Table 1). The study has been approved by the regional ethics committee in Linköping, Sweden (Dnr 2016/135–31; 2016/526–32; 2017/45–32). Recruitment started April 6, 2017 (Fig. 1) and is planned to continue until the 140 participants have been recruited.

**Table 1:** Schedule of enrolment, interventions, and assessments

	STUDY PERIOD					
	Enrolment	Allocation	Post-allocation			Close-out
			*t*_*1*_	*t*_*2*_	*t*_*3*_	*t*_*x*_
TIMEPOINT^c^	*0*	0				
ENROLMENT						
Eligibility screen	X					
Informed consent	X					
Allocation		X				
INTERVENTIONS^a^						
NSE			X	X	X	
NSEIT			X	X	X	
ASSESSMENTS^b^						
Demographic data	X		X			
Self-reported outcomes (questionnaires)			X	X	X	X
Clinical outcome			X	X	X	X

^a^NSE = 12 weeks of neck-specific exercise at physiotherapy clinic, NSEIT = 12 weeks of neck-specific exercise with 4 appointments at physiotherapy clinic + Internet support

^b^Questionnaires on various aspects of disability, pain, psychosocial factors, etc., including the main outcome the Neck Disability Index. Clinical outcomes including neck muscle endurance, range of motion, neurological tests, etc.

^c^t_1_ = baseline, t_2_ = 3-month follow-up, t_3 =_ 15-month follow-up, t_x =_ 15-month follow-up (12 months post-intervention)

### Study population

Individuals with a whiplash injury from a traffic accident with a four-wheeled motor vehicle at least 6 months ago but less than 5 years ago will be included after written and oral informed consent if they have chronic neck problems corresponding to WAD grades 2–3 [7] verified by clinical examination and have not participated in a neck-specific exercise programme in a previous research study [18, 19]. Additional inclusion criteria are: average estimated pain in the last week at least 20 mm on the visual analogue scale (VAS) [70], neck disability of more than 20% on the Neck Disability Index (NDI) [71], working age (18–63 years), daily access to a computer/tablet/smart phone and Internet, time to follow the treatment programme, and neck symptoms within the first week after the injury (i.e., neck pain, neck stiffness, or cervical radiculopathy).

Individuals with any of the following signs of head injury at the time of whiplash injury will be excluded: loss of consciousness, amnesia before or after the injury, altered mental status (e.g., confusion, disorientation), focal neurological changes (changes in smell and taste). Additional exclusion criteria are previous fractures or dislocation of the cervical column; known or suspected serious physical pathology included myelopathy, spinal tumours, spinal infection, or ongoing malignancy; previous severe neck problems that resulted in sick leave for more than a month in the year before the current whiplash injury; surgery in the cervical column; generalized or more dominant pain elsewhere in the body; other illness/injury that may prevent full participation in the study and/or in which neck exercises are contraindicated; inability to understand and write in Swedish; diagnosed severe mental illness, such as psychosis, schizophrenia, personality disorders; or current alcohol and drug abuse.

### Recruitment and randomization

Information on the study will be provided by healthcare providers, advertising in newspapers, posters, social media, and the university’s website. Patients may also be recruited consecutively as they seek treatment for their problems in primary healthcare. Interested patients will contact the research team (experienced physiotherapists) through the website. After completing a small survey on the website, a project team member will perform a telephone interview and ask about the patient’s medical history. As a last step an appointment for a physical examination is made to ensure the criteria for study participation are met. If the study criteria are met and written and oral informed consent obtained (distributed by the independent test leader), the patient will fill out a questionnaire (baseline data) and undergo physical measurements of neck-related function. Baseline measurements must be completed for inclusion.

A computerized block randomization list stratified by gender (conducted by a statistician and allocated by a project team member) will be used for randomization into two groups. Both groups will perform neck-specific exercises for 12 weeks: NSE (exercises at a physiotherapy clinic 2 times/week) or NSEIT (Internet-based support exercises in combination with four visits to the physiotherapist). The randomization will be performed by an independent researcher not otherwise involved in any of the tests or treatments. The researcher sends an opaque envelope containing the name and contact details of the patient and information about the randomization group to the treating physiotherapist, who calls the patient in to a new clinical examination (according to law) before treatment can start. The physiotherapists (in primary healthcare or working privately in out-patient care) will receive oral and written information and a day of practical training by the project leader. The treating physiotherapist is also able to consult with the project managers at any time if necessary. An exercise diary is maintained by patients in both groups and the number of care contacts recorded by the physiotherapist. A total of 140 participants with chronic WAD grades 2 and 3 will be recruited (70 per group).

### Intervention

In the NSE group, participants will get an explanation and justification for the exercise consisting of basic information about the musculoskeletal anatomy of the neck relevant to the exercises given by the physiotherapist in order to motivate the patient and help make them feel safe and reassured. Elements of a behavioural approach are also included, such as neurophysiological and neurobiological education and strategies for dealing with neck pain relapse. The patients undergo a 12-week training programme with a physiotherapist 2 days/week (total 24 times). Exercises are chosen from a clear and written frame of exercises. The training includes exercises for the deep neck muscles, continuing with the endurance training of neck and shoulder muscles. The exercises are individually adjusted according to the individual’s physical conditions and progressively increased in severity and dose. Exercise-related pain provocation is not accepted. The individual may also perform exercises at home. At the end of the treatment period, the participants are encouraged to continue practising on their own. The first visit to the physiotherapist is estimated to take 60 min and the others 30 min. The exercises have been used with good results in a previous RCT [18, 19] (DOI 10.3384/report.diva-113,865).

In the NSEIT group, participants will receive the same information and training programmes as the NSE group, but with four visits to the physiotherapist instead of 24. Exercises are introduced, progressed, and followed up to ensure correct performance. The exercises are performed and most of the information given with the help of Internet support outside the healthcare system. Photos and videos of the exercises, information, and answers to frequently asked questions are available on the Internet (Web-based system designed by the project leaders at the university). Patients can contact his/her physiotherapist if necessary. The first visit to the physiotherapist is estimated to take 60 min and the other visits 30 min. The time required for training is the same as in group A, but without the patient having to go to the physiotherapy clinic. The Internet programme was developed together with technicians, clinicians, and researchers and has been tested by people with chronic whiplash without any negative comments, except one person who had firewalls that needed to be adjusted on her/his work computer. Technicians are available to assist the participants should any technical difficulties arise, and patients receive follow-up questions regarding the Web support at the end of the intervention. The participants will learn the exercises and get information and support at the physiotherapy visits.

### Variables and measurements

All questionnaires will be answered electronically through a website. Participants will receive a disposable code e-mailed by a project team member to log in to the system (not the first survey before study inclusion). The electronic questionnaire cannot be submitted if the core outcomes are not answered. If a participant does not answer the questionnaire after two reminders (1.5 weeks after it is due and after another 1.5 weeks), a project team member not involved in the randomization procedure will phone the patient to collect the core outcomes of the NDI and pain variables. The Web-based questionnaire has been tested by both the project group and people with chronic neck pain (including WAD) and adjusted thereafter. Clinical measurements will be performed by test leaders in primary care settings in the different counties involved in the study.

Background data that will be collected include age, sex, family status, symptom duration, former healthcare, education, occupational classification (“Standard for Swedish work classification” SSY code [72]), post-traumatic stress reactions using the Post-traumatic Stress Disorder checklist (PCL-S) [73, 74], and the expectation of future treatment.

The primary outcome measure that will be collected is neck-specific function as measured by the NDI [71] (for example of the data collection form, please see http://www.aaos.org/uploadedFiles/NDI.pdf).

Secondary outcome measures that will be collected include neck-related function as measured by the Whiplash Disability Questionnaire [75–77] and patient-specific functional scale [78]; pain intensity in the neck, head, and arm (0–100 mm VAS) [70]; distribution of pain (Pain Sketch app) [79]; frequency of pain as measured by a 5-grade scale and use of pain medications; dizziness/balance according to the Dizziness Handicap Inventory (DHI) [80] and VAS [70]; headache according to a VAS [70] and the Headache Impact Test (HIT-6) [81]; cognitive ability according to the Cognitive Failures Questionnaire (CFQ) [82]; catastrophizing according to the Pain Catastrophizing Scale [83]; confidence in ability (Self-Efficacy Scale) [84]; operating fear according to the Fear Avoidance Beliefs Questionnaire (FABQ) [85, 86]; depression and anxiety measured with the Hospital Anxiety and Depression Scale (HAD) [87]; self-rated work ability measured by the Work Ability Index (WAI) [88–90]; sick days recorded (number of days/part of days, and dates of periods of sick days according to the Social Insurance Agency MIDAS register); income according to the Swedish tax office; health-related quality of life (EuroQuol) [91]; Global Rating of Change Scale [92]; requirements-effort support in the workplace according to the Effort Reward Imbalance [93]; ergonomics and how work is perceived; sickness presence (Stanford presenteeism scale) [94, 95]; self-assessment of sick-leave and data from the social security office; expectations and satisfaction with healthcare fulfilled by Cherkin Symptoms Satisfaction [96] and Patient Enablement Instrument [97] and questions of how they perceived study participation; level of physical activity measured with two questions about daily activities and sports [98, 99]; and adherence to treatment according to an exercise diary. Self-assessed care consumption and data obtained from the county council’s healthcare database or similar will be used in the cost-effectiveness analysis, and self-assessed consumption of analgesic drugs and data from the drug registry will also be collected. Two reminders will be send to participants who do not answer the questionnaires.

Tests that will be performed by the test leader [99–106], an independent physiotherapist, include active and passive range of neck motion [100], neck muscle endurance [99], cervical flexion-rotation test [101], palpation for segmental tenderness in the upper cervical spine [102], sensorimotor control [103], balance standing on one leg with eyes closed [104], hand strength [105], and neurology related to the neck, such as sensibility (touch and pin prick), upper limb reflexes, segmental identification muscle strength, upper limb tension test, Spurling’s test, and traction test [106].

In addition to being an outcome measure, physical examinations were used in combination with medical history for WAD grading [7] and potential additional diagnosis of cervicogenic headache [107]. Any important harms or unintended side effects in each group will be collected by the test leaders.

### Ethical considerations

The study was approved by the regional ethical review board in Linköping, Sweden. A previous clinical study relating to three different exercise interventions for long-term problems after whiplash injury [10, 11, 18–20] demonstrated good efficacy of neck-specific exercise performed in a primary healthcare setting. Current treatment is according to the best scientific evidence, and exercises used in the present study have been used in daily clinical practice for decades in the rehabilitation of various forms of neck pain. All of the physical tests are well-established and used both clinically and in previous research studies, are non-invasive, and do not cause danger or harm to the individual except for some risk of muscle soreness. Participants are included after a thorough clinical examination and provide signed informed consent. The exercises are adjusted individually. Participants are insured by the Swedish Legal, Financial, and Administrative Services Agency (Kammarkollegiet).

Login is required to fill in Internet-based surveys and to gain access to the Internet-based exercise programme. If the effect of the Internet-based exercise programme is good, the researchers’ intention is that it be used in clinical practice. People with long-term problems often have little access to assessments and treatments, as more acute conditions are often prioritized in the healthcare system. Both groups in the present study get active care that is expected to increase work ability. The benefit is deemed to be great, and there are no commercial interests.

The results will be presented at the group level, and no connection to the individual person can be made. All data are anonymous and subject to the official health secrets act.

### Statistical analysis

Sample size and power regarding group differences were calculated by a statistician (non-inferiority trial, an assumption that treatment B is not inferior to A) in the software PASS (version 13.0.8) based on the primary outcome NDI [71]. To detect a clinically relevant improvement of 7% in the NDI, 47 participants are needed in each group for 80% power. For non-inferiority tests, the significance level was set to 5% (*p* > 0.05), which corresponds to the one-sided confidence interval (95% CI). To be improved, both groups need a 7% increase in NDI (mean or median value depending on data) [71, 108, 109]. A non-inferiority border of less than 7% was chosen for mean NDI scores because it is on the border of what would be considered a clinically important effect [71, 108]. In addition, for secondary outcomes, the mean/median score will be on the border of what would be considered a clinically important effect for each measurement. The 95% CI will be examined and, if the upper limit of the interval is less than the chosen border value, non-inferiority of NSEIT to NSE will be concluded. For background data, and if appropriate after the non-inferiority test, a two-sided superiority test will be performed with a significance level of 5%. These values are based on the standard deviation (SD 13.4) of the NDI in a previous study of neck-specific exercises in individuals with chronic WAD grade 2 and 3 [18, 19]. To ensure that enough people are in each group after drop-outs, for prediction analyses, and the opportunity for subgroup analyses, 70 participants will be included in each group.

Analyses will be performed in collaboration with a statistician using parametric or non-parametric statistics depending on the type of data and whether the analysis is between groups or over time. Analyses will be performed primarily on an intention-to-treat basis (as individuals being randomized into the two groups) and secondarily on a per protocol basis (counted on individuals who fulfilled the programme for at least 50%). Imputation methods may be used when deemed to have additional value. Subgroup analyses of age, gender, WAD grade, headache, and dizziness may possibly be performed. Database monitoring will be performed by the project leaders and statisticians involved after study completion, independent of sponsors and competing interests. The project leaders and collaborating statisticians and researchers will have access to the final trial dataset (after the project leaders’ allowances).

Background data will be evaluated by descriptive statistics and differences in baseline data determined using t-test (mean and standard deviation) or non-parametric test where appropriate. A linear mixed model or general linear mixed model (GLMM) may be used depending on the data. NDI will be evaluated as a dependent variable with independent fixed factors time (baseline, 3 months, and 15 months after start of intervention) and group (A and B). If the non-inferiority of B to A is concluded based on the 95% CI, a test of the superiority of B to A will be performed as suggested by Lessafre [110]. Cost-effectiveness will be calculated from both a societal and healthcare perspective using incremental cost-effectiveness ratios (ICERs) based on the EQ-5D for QALY calculations. The variation in response to intervention (heterogeneity of treatment effect) will be evaluated using regression analysis.

### Timetable

The project started April 6, 2017. The inclusion period is estimated to be finalized after approximately 2 years. Thereafter, participants will be followed for another 15 months. Ethical approval has been obtained.

### Plan for the implementation and dissemination of research results

Several factors can hinder or facilitate the introduction of scientific knowledge into practical or organizational activities. These factors may be related to the individual caregiver, the social context of the caregiving environment, or the organizational context. A survey of these factors will provide direction for implementation and a basis for selecting the best implementation strategy. Educational sessions are commonly used to implement new treatment strategies. Previous research of such sessions has established that the inclusion of interactive components makes them more successful. Other important determinants of success are innovation characteristics (e.g., neck-specific training), adopter characteristics, implementation strategies, and contextual factors. The long-term goals of this study are to optimize treatment plans for patients, which will improve the rate at which they can return to work and participate in society.

## Discussion

The clinical advantages of the project are great because patients with long-standing WAD experience disability at great personal and social cost. Thus, an urgent need exists to expand our knowledge of rehabilitation. Areas to be explored include disability associated with WAD and the effect of Internet-based physiotherapy treatment outcomes. The present study will help provide evidence of treatment efficacy for patients with WAD. The long-term goals of this study are to optimize treatment plans for patients, which will improve the rate at which they can return to work and participate in society.

## Trial limitations

This is a multicentre study involving multiple intervening therapists, which offers less control, and the performance of the intended interventions may be compromised. However, to minimize this risk, the physiotherapists will be trained by the project leaders and have time to practise the standardized interventions in preparation for the study. Furthermore, a multicentre study may be more generalizable and enhance implementation. Participants need to have access to a computer/smartphone/tablet and an Internet connection. Most people, even those in the older age groups, have access to such equipment nowadays [59]. To be able to answer the questionnaires and understand the Web-based programme, participants need to speak and read Swedish. However, providing that the results of the Web-based programme will be equally good, it can be translated into other languages to include a non-Swedish-speaking population.
